# How can functional annotations be derived from profiles of phenotypic annotations?

**DOI:** 10.1186/s12859-017-1503-5

**Published:** 2017-02-10

**Authors:** Beatriz Serrano-Solano, Antonio Díaz Ramos, Jean-Karim Hériché, Juan A. G. Ranea

**Affiliations:** 1 Department of Molecular Biology and Biochemistry, University of Málaga, Boulevard Louis Pasteur, Málaga, 29071 Spain; 2 Department of Algebra, Geometry and Topology, University of Málaga, Boulevard Louis Pasteur, Málaga, 29071 Spain; 3 European Molecular Biology Laboratory, Meyerhofstrasse 1, Heidelberg, 69117 Germany; 4 CIBER de Enfermedades raras (CIBERER), Madrid, Spain

**Keywords:** Ontology, Cellular phenotype, Biological network, Cluster analysis

## Abstract

**Background:**

Loss-of-function phenotypes are widely used to infer gene function using the principle that similar phenotypes are indicative of similar functions. However, converting phenotypic to functional annotations requires careful interpretation of phenotypic descriptions and assessment of phenotypic similarity. Understanding how functions and phenotypes are linked will be crucial for the development of methods for the automatic conversion of gene loss-of-function phenotypes to gene functional annotations.

**Results:**

We explored the relation between cellular phenotypes from RNAi-based screens in human cells and gene annotations of cellular functions as provided by the Gene Ontology (GO). Comparing different similarity measures, we found that information content-based measures of phenotypic similarity were the best at capturing gene functional similarity. However, phenotypic similarities did not map to the Gene Ontology organization of gene function but to functions defined as groups of GO terms with shared gene annotations.

**Conclusions:**

Our observations have implications for the use and interpretation of phenotypic similarities as a proxy for gene functions both in RNAi screen data analysis and curation and in the prediction of disease genes.

**Electronic supplementary material:**

The online version of this article (doi:10.1186/s12859-017-1503-5) contains supplementary material, which is available to authorized users.

## Background

A central tenet of experimental approaches to assigning functions to genes posits that genes involved in the same biological process show similar loss-of-function phenotypes. This provides the rationale for performing loss-of-function genetic screens and is used by the Gene Ontology consortium in their gene annotation process (i.e. for annotations with evidence code IMP: Inferred from Mutant Phenotype, The Gene Ontology Evidence Tree^1^). Systems microscopy approaches, defined as the combination of recent developments in microscopy automation with automated image analysis and data mining [1], now allow for systematic exploration of the gene loss-of-function phenotypic space and large scale RNAi screens have given us phenotypic information for thousands of genes (e.g. [2–4]). In contrast to more traditional experiments that have been addressing a single phenotype closely associated with a function, systems microscopy approaches increasingly use phenotypic profiling, the description of phenotypes by multi-parameter measurements. While this increases the amount of usable information, the cost is that functional associations become less evident. The process of converting phenotypic annotations to functional annotations therefore remains a manual one, due to the free-text nature of many phenotypic descriptions and to the difficulty of assessing phenotypic similarity (i.e. how similar should two phenotypes be in order to infer the same function?) in particular across different experiments. As a consequence, large RNAi screens performed in human cells haven’t been used to annotate genes with Gene Ontology terms from the biological process domain and this contributes to a lower level of experimentally-supported annotations of genes cellular functions than the number of reported functional assays. In this context, automating the identification of similar cellular phenotypes and their assignment to relevant cellular processes across experiments would increase the level of experimentally-supported functional annotations. The recently developed Cellular Phenotype Ontology (CPO, [5]) and Cellular Microscopy Phenotype Ontology (CMPO, [6]) attempt to fill a gap in the domain coverage of existing ontologies by organizing the cellular phenotype domain into a consistent ontological structure. Replacing free-text phenotypic description in RNAi screens with well-defined ontology terms makes automatic evaluation of phenotypic similarity possible. However, to automatically convert phenotypic annotations, in particular phenotypic profiles, to functional annotations, we need to understand how phenotypes and functions are related. As most screens report hit lists usually found enriched in genes involved in relevant biological processes using GO annotations, we can expect phenotypic similarity to correlate with or be indicative of participation in similar cellular processes. We wondered how phenotypic profiles generated by combining annotations from multiple screens could be exploited to automate and/or refine gene functional annotations. Note that our goal is not to remine the screens to infer gene function but rather to explore whether and how the phenotypic annotations resulting from these screens can be related to Gene Ontology biological process terms.

## Methods

### Gene phenotypes

Gene loss-of-function phenotypes were obtained from the following large siRNA-based gene silencing experiments performed in human cells: Mitocheck [2], EMBL secretion screen [4], EMBL chromosome condensation screen [7], Copenhagen DNA damage screen [8], CellMorph [3]; and RNAi screens GR00290-A (regulation of centriole biogenesis, [9] and GR00053-A (genome stabilization by phosphorylation of the histone H2AX, [10] from the GenomeRNAi database [11]. It is noteworthy that none of these screens have been used for making biological process annotations in GO (as evidenced by the fact that none of the corresponding papers are cited as source of annotation) despite the data having been available for several years. The cellular functions covered by these screens are diverse and include cell proliferation, cell death, cell motility, mitosis, protein secretion, DNA damage and centriole formation. However, this list is not exhaustive as some screens (e.g. CellMorph, MitoCheck) report phenotypes not obviously associated with a declared target biological function.

The compilation of all data from these separate experiments, gives a set of 36 unique cellular phenotypes (Table 1) associated with 4198 Entrez genes (see Additional file 1: Table S1). Most genes have been tested in more than one screen, and the screens include non-overlapping sets of phenotypes. As our goal is to explore how phenotypic annotations are linked to GO cellular process terms, we used phenotypic annotations resulting from the screens as made available in the corresponding papers. Relationships between genes and phenotypes from different assays were integrated into a binary matrix recording the presence (value 1) or absence (value 0) of a phenotype for each gene (Table 2). Note that 0 is also used where genes have not been tested in a screen. To assess whether this affected our results, we tested the effect of sparsity by replacing a proportion (5, 10, 20 and 30%) of randomly selected 1 s with 0 s. A visual overview of the data matrix is presented in Additional file 2: Figure S1.

**Table 1:** Set of 36 phenotypes obtained from the listed siRNA experiments sorted by its CMPO identifier

Experiment	Description	Phenotypes	IDs in CMPO
CellMorph [3]	Genome-wide RNAi screen	Decreased cell number cell with projections	CMPO:0000052
	that examines changes in	elongated cell more lamellipodia cells increased	CMPO:0000071
	the morphology of	number of actin filament round cell increased cell	CMPO:0000077
	individual HeLa cells within	size decreased cell size bright nuclei metaphase	CMPO:0000083
	cell populations.	arrested increased cell size in population	CMPO:0000105
			CMPO:0000118
			CMPO:0000128
			CMPO:0000129
			CMPO:0000154
			CMPO:0000305
			CMPO:0000340
MitoCheck [2]	Genome-wide RNAi screen	Cell death increased nucleus size graped	CMPO:0000030
	for genes required for	micronucleus abnormal nucleus shape mitosis	CMPO:0000140
	chromosome segregation in	delayed binuclear cell absence of mitotic	CMPO:0000156
	HeLa cells. The screen also	chromosome decondensation increased cell	CMPO:0000157
	reports genes involved in	movement speed increased cell movement	CMPO:0000202
	other processes such as cell	distance proliferating cells metaphase delayed	CMPO:0000213
	movement.	abnormal chromosome segregation prometaphase	CMPO:0000216
		delayed increased variability of nuclear shape in	CMPO:0000236
		population mitotic metaphase plate congression	CMPO:0000237
			CMPO:0000241
			CMPO:0000307
			CMPO:0000326
			CMPO:0000344
			CMPO:0000345
			CMPO:0000348
EMBL secretion [4]	Genome-wide RNAi screen	Increased rate of protein secretion mild decrease	CMPO:0000246
	for interference with	in rate of protein secretion strong decrease in rate	CMPO:0000318
	ER-to-plasma membrane	of protein secretion decreased rate of intracellular	CMPO:0000319
	transport of the secretory	protein transport	CMPO:0000346
	cargo protein tsO45G in		
	HeLa cells.		
GR00053 [10]	Genome-wide RNAi screen	Increased number of site of double-strand break	CMPO:0000182
	for genes involved in DNA		
	damage responses in HeLa		
	cells.		
GR00290 [9]	Genome-wide RNAi screen	Increased centriole replication decreased	CMPO:0000361
	for genes regulating	centriole replication	CMPO:0000362
	centriole formation in HeLa		
	cells.		
Copenhagen DNA damage Ubiquitin [8]	RNAi screen of >1300	Decreased number of site of double-strand break	CMPO:0000181
	genes involved in the		
	ubiquitin-proteasome		
	system or encoding		
	zinc-finger proteins looking		
	for modulators of cellular		
	responses to ionizing		
	radiation in HeLa and		
	U2OS cells.		
EMBL chromosome condensation [7]	RNAi screen of 100	Increased duration of mitotic prophase decreased	CMPO:0000328
	bioinformatically-selected	duration of mitotic prophase	CMPO:0000329
	genes for changes in mitotic		
	prophase duration in HeLa		
	cells.

**Table 2:** Binary matrix for gene-phenotype association

Gene	Decreased	Cell with	…	Mitotic metaphase
	cell number	projections		plate congression
	(CMPO:0000052)	(CMPO:0000071)		(CMPO:0000348)
57147 (SCYL3)	1	0	…	0
2268 (FGR)	1	0	…	1
22875 (ENPP4)	0	1	…	0
…	…	…	…	…
5439 (POLR2J)	1	0	…	1

Presence and absence of a phenotype after inhibition of each gene is represented by values 1 and 0, respectively

### Ontologies and annotations

**Fig. 1:**
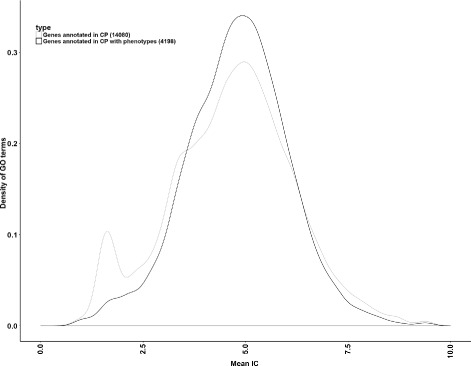
Distribution of information content (IC) of the terms annotating genes with phenotypes (*black*) and all the terms in cellular process (*grey*). For each level of specificity represented by the information content (IC), the *curves* represent the proportion of genes annotated with terms of this level in all the annotated genes versus the subset of genes with phenotypes

We used two formal ontologies to perform our study: the Gene Ontology (GO) [12] and the Cellular Microscopy Phenotype Ontology (CMPO)^2^. We selected for our study the GO branch of cellular process (root term GO:0009987), which is the ontological domain closer to the cellular phenotypes captured in the screens. The terms hierarchy was extracted from the OBO file released on 2015-09-26. Gene Ontology annotations of genes were downloaded from the GO web site^3^ (see Additional file 3: Table S2) and extracted from the file with validation date: 09/16/2015, removing electronically-inferred annotations (IEA). To ensure that the genes with phenotype did not form a biased set of GO annotations, we verified that the distribution of information content of the terms used to annotate the genes with phenotypes was the same as for all annotated genes (Fig. 1).

CMPO gene annotations were retrieved from the cellular phenotype database^4^ [13](see Additional file 4: Table S3). Compared to a vocabulary of phenotypes, the ontology has the advantage of formalizing the relationships between the phenotypes. For example, the ontology allows to infer that the phenotypes “chromosome segregation defect” and “metaphase arrest” are both mitotic phenotypes.

### Similarity measures

Similarity measures used in this study are shown in Table 3. Euclidean and correlation distances were computed using the R core package *stats*, for cosine we used *lsa* [14] and for Jaccard *prabclus* [15]. Hamming, Cohen’s kappa and TF-IDF [16] were also coded in R. For dimensionality reduction, we applied the *logisticPCA()* function of the R package *logisticPCA* to extract 10 principal components and correlation, cosine and Euclidean similarities were computed in this new space. To take advantage of the phenotype ontology, we also computed several measures of semantic similarity using the R package *dnet* [17].

**Table 3:** Similarity measures used in this study

Name	Formula
Euclidean similarity	$s^{2}\left (g_{1}, g_{2}\right)=\frac {1}{1+\left (x_{g1}-x_{g2}\right)\left (x_{g1}-x_{g2}\right)^{'}}$
Correlation similarity	$s\left (g_{1},g_{2}\right) = \frac {\left (x_{g1}-\overline {x}_{g1}\right)\left (x_{g2}-\overline {x}_{g2}\right)^{'}} {\sqrt {\left (x_{g1}-\overline {x}_{g1}\right)\left (x_{g1}-\overline {x}_{g1}\right)^{'}} \sqrt {\left (x_{g2}-\overline {x}_{g2}\right)\left (x_{g2}-\overline {x}_{g2}\right)^{'}}}$
	where $\overline {x}_{g1}=\frac {1}{n}\sum _{p \in P}x^{p}_{g1}$ and $\overline {x}_{g2}=\frac {1}{n}\sum _{p \in P}x^{p}_{g2}$
Cosine similarity	$s\left (g_{1},g_{2}\right) = \frac {x_{g1}x_{g2}^{'}}{\sqrt {x_{g1}^{'}x_{g1}} \sqrt {x_{g2}^{'}x_{g2}}}$
Hamming similarity	$s\left (g_{1},g_{2}\right) = \frac {x^{p}_{g1}=x^{p}_{g2}}{n}$
Jaccard similarity	$s\left (g_{1},g_{2}\right) = 1 - \frac {\left [\left (x^{p}_{g1} \neq x^{p}_{g2}\right)\wedge \left (\left (x^{p}_{g1} \neq 0\right) \vee \left (x^{p}_{g2} \neq 0\right)\right)\right ]} {\left (x^{p}_{g1} \neq 0\right) \vee \left (x^{p}_{g2} \neq 0\right)}$
Cohen’s kappa	$s\left (g_{1},g_{2}\right)=\frac {p_{0}-p_{c}}{1-p_{c}}$ where:
	- *p*_0_ is the proportion of terms common to profiles *g*_1_ and *g*_2_, and
	- *p*_*c*_ is the proportion of terms common to profiles *g*_1_ and *g*_2_ expected by chance.
TF-IDF similarity	$s\left (g_{1},g_{2}\right) = \max _{p \in P}\left \{x^{p}_{g1}x^{p}_{g2}IDF(p)\right \}$ where$IDF(p)=log\frac {n_{G}}{1+\sum _{g \in G}{x^{p}_{g}}}$
Resnik’s semantic similarity	*s*(*t*_1_,*t*_2_)=*IC*(*t*_*MICA*_) where:
	- the Most Informative Common Ancestor is$t_{MICA}={argmax}_{t \in S\left (t_{1},t_{2}\right)}{IC(t)}$,
	- the information content (IC) of a term *t* is *IC*(*t*)=−*log*(*p*(*t*)),
	- the probability of a term *t* is $p(t)=\frac {annotations(t)}{totalAnnotations}$, and
	- *S*(*t*_1_,*t*_2_) is the set of common ancestors of *t*_1_ and *t*_2_.
Lin’s semantic similarity	$s\left (t_{1},t_{2}\right) = {\frac {{2\cdot IC\left (t_{MICA}\right)}}{IC\left (t_{1}\right)+IC\left (t_{2}\right)}}$
Schlicker’s semantic similarity	$s\left (t_{1},t_{2}\right) = \frac {2\cdot IC\left (t_{MICA}\right)}{IC\left (t_{1}\right)+IC\left (t_{2}\right)}\cdot \left (1-p\left (t_{MICA}\right)\right)$
Jiang’s semantic similarity	*s*(*t*_1_,*t*_2_)=1+2·*IC*(*t*_*MICA*_)(*IC*(*t*_1_)+*IC*(*t*_2_))
Pesquita’s semantic similarity	$s\left (t_{1},t_{2}\right) = \frac {\sum \limits _{t \in S(t_{1},t_{2})}{IC(t)}}{\sum \limits _{t \in P(t_{1},t_{2})}{IC(t)}}$ where:
	- *P*(*t*_1_,*t*_2_) is the set of ancestors of either *t*_1_ or *t*_2_.

*G* is the full set of genes (*n*_*G*_=4198) and *P* is the set of 36 (*n*_*P*_) phenotypes. *x*_*g*_ denotes the phenotypic profile of gene *g* with $x^{p}_{g}=1$ if *g* shows phenotype *p*, $x^{p}_{g}=0$ otherwise

### Comparison of phenotypic similarity measures

To evaluate how the similarity measures related to each other, similarities between pairs of genes were computed for each measure. Pearson’s correlation coefficient (PCC) between the measures was then computed from these sets of values. Hierarchical clustering was performed by average linkage using the *hclust* R package with 1-PCC as distance measure.

To rank the similarity measures in relation to their ability to capture gene function we used protein interaction as a proxy for functional relationships between genes. To this end, we first ranked the similarity measures by their ability to distinguish between interacting and non interacting gene pairs using the area under the ROC curve (AUC). In this context, the AUC can be interpreted as the probability that the similarity measure ranks an interacting gene pair higher than a non-interacting one. As positive interacting pairs, we used physical protein interactions from Intact [18], MIPS [19], DIP [20] and BIOGRID [21] that have been reported by two different experimental methods and curated interactions from Reactome [22, 23]. As negative interactions, we used the curated negative interactions from the MIPS Negatome [24] and Trabuco et al. [25]. The AUCs were computed using the R package *pROC* [26].

We also computed a score for each measure as the number of genes whose most phenotypically similar gene is also an interaction partner in the iRef index protein interaction data (release 14.0, April 7th, 2015) [27]. For each measure, the nearest neighbor of each gene was identified (ties were broken at random) and the measure’s score was incremented by one if the two genes formed a known interacting pair in the iRef index. To assess the statistical significance of the score, the probability of having the same or better score from a random selection of protein interactions was computed from the hypergeometric distribution using the *phyper()* function in R as follows: We considered 4198 genes making a total of 4198∗(4198−1)/2 possible interactions of which 29649 were present in iRef index. For a given measure of similarity, we tested 4198 interactions (one for each gene). Therefore, the probability of having a score of x or better by selecting the interactions randomly is given by 1−*phyper*(*x*−1,29649,4198∗(4198−1)/2−29649,4198).

### Annotation-driven approach

Following the approach by Glass and Girvan [28], a bipartite graph was constructed, for functions and phenotypes respectively, by setting an edge between two GO terms (resp. CMPO terms) if they shared at least a gene and the edge was weighted by the number of genes shared. Because high level terms inherit genes from their child terms, term degrees are biased. To compensate for this, we normalized edge weights by the union of the genes belonging to the two terms. We then grouped terms by spectral clustering using the normalized cut objective function [29] with an arbitrary number of clusters, set to 13 for CMPO and 140 for GO. GO terms clusters were obtained by first partinioning the graph into 100 clusters then partitioning again the two largest clusters into 33 and 9 clusters. As noted by Glass and Girvan [28], different numbers of clusters correspond to different levels of specificity. We chose the number of GO terms clusters so that most clusters would be linked to phenotypes. The number of CMPO terms clusters was chosen to produce a reasonable distribution of cluster sizes minimizing the number of clusters with only one single term. Increasing the number of clusters leads to an increase in the number of clusters containing only one term.

### Correction for multiple testing

*P*-values were corrected for multiple testing using the R function *p.adjust()* with the Benjamini and Hochberg method.

## Results

### Comparison of phenotypic similarity measures

As we wished to link phenotypic similarity to gene function, the first question we addressed is which measure of phenotypic similarity to use for the task. Similarity between phenotypic profiles has typically been assessed using feature vector-based similarity measures such as correlation [30, 31] or cosine (e.g. [32, 33]). Due to their binary nature, profiles can also be compared using character-based (binary) similarity measures. For example, the main component of the PhenoBlast algorithm for retrieving profiles similar to a query [34] is the number of matches in the binary string. PhenoBlast also recognizes that some phenotypes may be more informative than others and one of its components is the probability of observing a given combination of shared phenotypes by chance. Combining these two components into one measure leads to Cohen’s kappa measure of similarity between two profiles. The intuition that some phenotypes are more informative than others can be formalized by using information content-based similarity measures. Here, information content refers to the specificity of a phenotype. Typically, a phenotype is considered more specific if it is less often observed e.g. cell death, a widely observed phenotype, is considered less specific than mitotic delay which is more rarely observed. This leads to TF-IDF similarity measures in which phenotypes are weighted by the inverse of their frequency of occurrence in the data [35]. The availability of CMPO now also allows for a semantic information content-based approach to phenotypic similarity, analoguous to what has been used with Gene Ontology annotations (e.g. Resnik’s similarity measure). In an ontology, the information content of a term also takes into account the structure of the ontology such that child terms are more specific than their parents. When working with feature vectors of high dimension, it is sometimes beneficial to compute vector-based similarity measures in a reduced dimensional space. As phenotypic profiles are high dimensional vectors, we also wondered if a dimensionality reduction approach would be beneficial and applied logistic PCA, an extension of standard PCA to binary data, to compute vector-based phenotypic similarities in a reduced dimensional space.

**Fig. 2:**
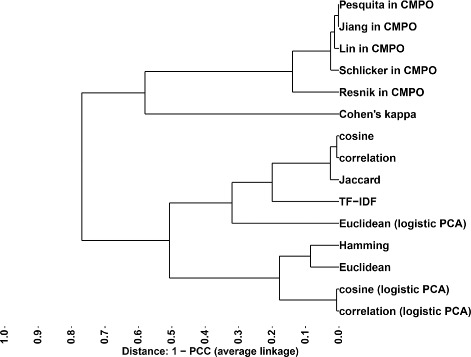
Hierarchical clustering of phenotypic similarity measures based on Pearson correlation distance

Given these different ways in which to measure phenotypic similarities, we wondered whether they were equivalent in ranking genes based on their phenotype profiles. To answer this question, we computed the correlation coefficient between phenotypic similarities obtained with the different measures and performed hierarchical clustering. The resulting dendrogram (Fig. 2) shows that the similarity measures fall into two main groups with the information content-based semantic similarity measures (Resnik, Schlicker, Lin, Jiang and Pesquita) distinctly separated from the feature vector-based measures (cosine, Euclidean, correlation, Jaccard and Hamming), with Cohen’s kappa occupying an intermediate position, confirming our intuition that these groups of measures assess phenotypic similarity in different ways. We next asked whether this difference was meaningful with respect to biological function. To test this, we used protein interactions as a proxy for biological function, i.e. two interacting proteins are taken as indication that the corresponding genes are involved in the same function [36]. This means that, for a relevant measure, phenotypically similar genes are expected to be enriched in protein interactions. We tested this in two ways. First, we assessed the ability of each measure to distinguish between interacting and non-interacting gene pairs by computing the area under the ROC curve (AUC) using high-confidence physical protein-protein interactions as positive set and curated non-interacting protein pairs as negative set. In this context, the AUC is the probability that the similarity measure ranks an interacting gene pair higher than a non-interacting one. A similarity measure with no discriminating power has an AUC of 0.5 and higher values indicates increasingly better discriminative power. Using this approach, the best similarity measures are Resnik’s and Schlicker’s with the other semantic similarity measures outperforming the character- and vector-based measures (Table 4). Therefore, using semantic similarity measures, phenotypic profiles of interacting genes are overall more similar than for non-interacting gene pairs.

**Table 4:** Similarity measures sorted by area under the ROC curve (AUC)

Measure	AUC	Protein interactions	*p*-value
Resnik in CMPO	0.56	24	0.0102
Schlicker in CMPO	0.56	12	0.7512
Lin in CMPO	0.55	11	0.8332
Cohen’s kappa	0.54	27	0.0015
Pesquita in CMPO	0.54	14	0.5494
Jiang in CMPO	0.54	11	0.8332
TF-IDF	0.53	25	0.0055
Euclidean	0.53	16	0.3433
correlation	0.52	22	0.0311
Hamming	0.52	21	0.0513
cosine	0.49	13	0.6545
Jaccard	0.49	13	0.6545
Euclidean (logistic PCA)	0.46	25	0.0055
correlation (logistic PCA)	0.45	19	0.1242
Cosine (logistic PCA)	0.45	14	0.5494

The second column represents the number of nearest neighbour gene pairs who are also protein interaction partners, and the third one, the *p*-values (computed from the hypergeometric distribution) that the number of observed interacting pairs is due to chance

In a second approach, for each similarity measure, we identified the nearest (i.e. most similar) neighbour of each gene and tested whether the two genes were known interaction partners. To compare the phenotypic similarity measures, we then ranked them by the number of interactions retrieved in this way (Table 4). With this approach, TF-IDF and Resnik’s similarity performed best. Other semantic similarity measures and most feature-based measures (Euclidean, Jaccard and cosine) were not better than a random selection of protein interactions indicating that these phenotypic similarity measures may not adequately capture functional relationships. Dimensionality reduction as obtained by logistic PCA did not improve performance of the vector-based measures indicating that linear combinations of phenotypes are unlikely to capture links to function. Therefore, across the two tests, Resnik’s similarity measure appears the most consistent at associating similar phenotypes with interacting proteins. Other semantic similarity measures may have been negatively influenced by the sparsity of the CMPO ontology due to their attempts at accounting for more of the ontology structure than Resnik’s measure. For example, Lin’s and Jiang’s measures are particularly sensitive to variations in the ontology structure because they take into account the density and the level of the terms whereas Resnik’s measure only considers the lowest common ancestor and is thus comparatively more robust.

### Relationship between GO cellular process annotations and phenotypes

**Fig. 3:**
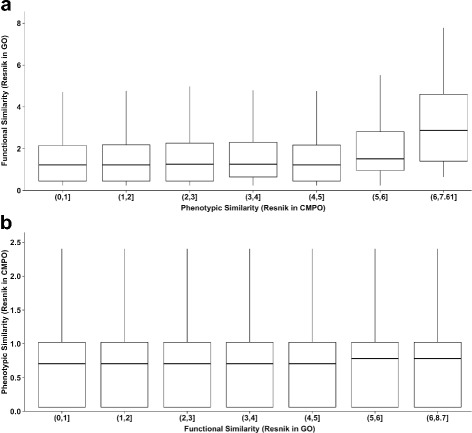
Distributions of functional and phenotypic similarities. The *box* represents the *upper* and *lower* quartiles and the median is represented by the *black* line inside the *box*. **a** Phenotypic similarity in CMPO versus functional similarity in GO. **b** Functional similarity in GO versus phenotypic similarity in CMPO

Having identified a suitable measure of phenotypic similarity, we set to explore how gene functions relate to phenotypes more directly. If phenotypes are predictive of biological functions, we expect that pairs of genes with similar phenotypes will have similar functions. Since gene functions have been standardized using the Gene Ontology, gene functional similarity was computed using Resnik’s semantic similarity between GO terms, a measure generally found to be the best for this purpose [37]. To assess links between gene phenotypic similarity and gene semantic similarity in GO, we plotted GO semantic similarities versus CMPO semantic similarities for the RNAi screen data (Fig. 3 a), excluding genes with no functional annotation in GO. The distribution of functional similarity values is the same for all levels of phenotypic similarity except the highest, which showed a trend towards higher functional similarity. Although weak, this effect is robust as it is still observed when removing up to 30% of the phenotypic annotations (see Additional file 5: Figure S2) and does not appear to be due to chance because random assignment of GO similarity values to high-scoring CMPO gene pairs resulted in a lower average GO similarity (Additional file 6: Figure 3). While this matched our expectation that specific phenotypes are associated with specific functions, this represented only a small fraction of the genes (20/4198) and for most genes, phenoypes do not appear to be good indicators of function.

One possible explanation for this result is that several functions could share the same phenotype. If that were the case, then we would predict that similar functions would still lead to similar phenotypes. We would then expect that two genes involved in the same cellular process would have similar phenotypes. However, this is not the case as genes with high functional similarity are not more likely to have high phenotypic similarity (Fig. 3 b). This lack of correlation between function and phenotype was also observed for the other phenotypic similarity measures tested, indicating that this was not an effect of the phenotypic similarity measure used. This effect is also observed when electronically-inferred annotations are included (see Additional file 6: Figure S3).

**Fig. 4:**
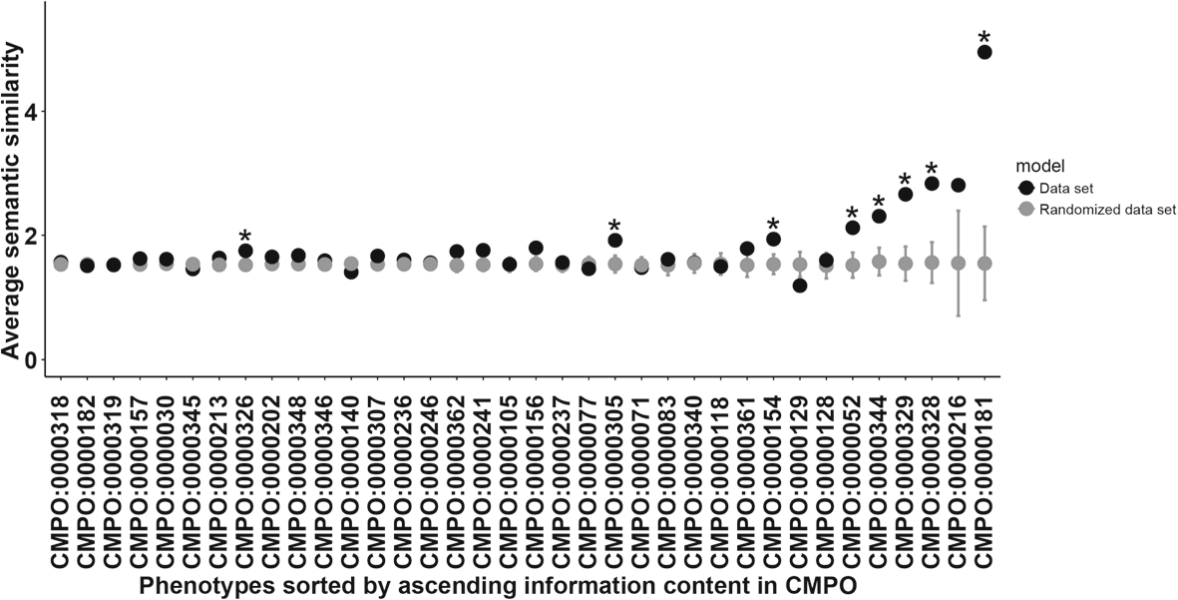
Average semantic similarity in GO between genes sharing a particular phenotype (*black*). Randomization of the relationships between phenotypes and genes represents the null model (*grey*). Phenotypes with genes having high functional similarity (FDR-corrected *p*-values ≤0.01) are marked with *. Phenotypes are sorted on the *X* axis by ascending information content in CMPO. CMPO descriptions for the identifiers are in Table 1

So neither considering the most informative phenotypic term nor the whole phenotypic profile gives any indication of function. This result is counter-intuitive since the premise of most screens is that genes with the same biological function would give the same loss-of-function phenotype or phenotypic profile. We hypothesized that perhaps even in screens which relied on profiling, each phenotype is individually indicative of a function. To test whether this also holds across screens, we averaged the semantic similarity in GO for all pairs of genes showing a particular phenotype. Then, we compared this average to that obtained from 100 datasets generated by randomly shuffling the associations between genes and phenotypes while keeping the number of links per phenotype unchanged. A total of 8 out of 36 (25%) phenotypes gave a statistically significant signal (FDR-corrected *p*-value ≤0.01) for having their actual functional similarity between genes above that obtained by randomization (Fig. 4). Half of these significant phenotypes correspond to CMPO terms with high information content indicating that only specific phenotypes tend to associate with highly similar GO functional annotations. While these results conform to our intuition, the only practical rule that can be derived for automatically converting phenotypic annotations to functional annotations is that only phenotypes with CMPO semantic similarity over some threshold are indicative of similar cellular function.

### Gene annotation-driven phenotypic and functional similarity

**Fig. 5:**
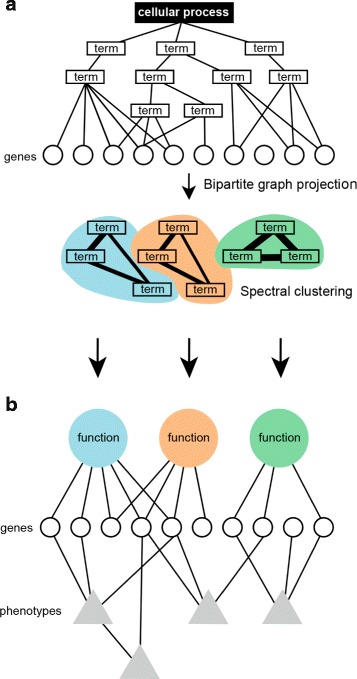
Annotation-driven cellular function definition. **a** Genes (*circles*) are annotated with cellular process ontology terms (*rectangles*). After bipartite graph projection, links between terms are weighted according to the number of genes shared (*line width*). Then, terms are grouped using spectral clustering. **b** Clusters of functional terms (*coloured circles*) are linked to phenotypes (*triangles*) by shared genes

**Fig. 6:**
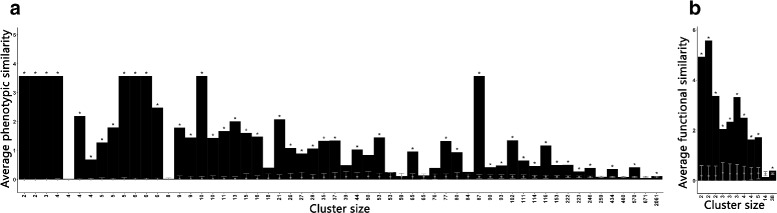
Average semantic similarity between terms in clusters. Randomization of the assignments of terms in clusters are represented in *grey*. Clusters are sorted by size (i.e. number of terms). **a** Average phenotypic similarity in clusters of GO terms. **b** Average functional similarity in clusters of CMPO terms

The above results suggested that the Gene Ontology structure does not adequately capture the functional relationships that underlie phenotypic similarity. An alternative way of organizing GO terms has recently been proposed by Glass and Girvan [28]. In this scheme, a term graph is generated by linking terms based on the genes annotated with them. Thus, two terms are more similar the more genes they share (i.e. the more genes are annotated with both terms). Biological functions can then be defined as groups of similar terms by applying a clustering algorithm to the term graph (Fig. 5). In this scheme, a function can be seen as being represented by a signature of co-occurring terms. We wondered if this approach would allow us to recover a broader relationships between functions and phenotypes. To test this, we grouped the cellular process GO terms into 140 clusters. To assess whether this new definition of function captured phenotypic similarity we computed Resnik’s similarity between CMPO terms associated with genes within each cluster (Fig. 6 a). Excluding functional clusters not linked to phenotypes, 77*%*(45/58) of functional clusters had high phenotypic similarity that could not be explained by chance assignment of GO terms to clusters (FDR-corrected *p*-value ≤0.01, Fig. 6 a). Therefore cellular functions derived from shared gene annotations were associated with phenotypic similarity. To test whether similar phenotypes reflected similar functions, we defined a phenotypic terms graph in the same way and grouped the phenotypes into 13 clusters. Each of these phenotypic cluster can be viewed as a phenotype characterized by a signature of co-occurring phenotypic descriptors. As above, for each phenotypic cluster, we computed Resnik’s similarity between GO terms within clusters. Again, except for clusters with no GO annotations, we observed that functional similarity was higher in phenotypic clusters than can be explained by random phenotype assignments to clusters (Fig. 6 b). This indicated that this definition of phenotype was able to recover functional similarity in GO. Therefore, functions defined by groups of GO terms sharing associated genes tend to map to CMPO terms better than functions defined by individual GO terms and conversely, phenotypes defined by groups of CMPO terms sharing associated genes map better to GO terms than phenotypes defined by individual CMPO terms. While the details of how phenotypes and functions are defined is subject to changes in both CMPO and GO, the strong association between phenotypes and functions is robust as it only depends on the annotated genes.

## Discussion

The large amount of cellular phenotypic annotations coming from high-throughput genetic screens represents a largely untapped source of information on gene function. Our aim was to understand how these phenotypes are related to gene function in the hope that principles could be derived for use in automatically converting published phenotypic annotations to functional annotations. Here, we used published cellular phenotypes from large scale RNAi screens in human cells that have been annotated with CMPO terms to explore how cellular phenotypes related to GO cellular functions. The first question we addressed was how to adequately measure phenotypic similarity such that phenotypic similarity would be correlated with functional similarity. We found that, in contrast to feature-based similarity measures, information content-based phenotypic similarity measures like Resnik’s semantic similarity were best at associating high phenotypic similarity with protein interactions, suggesting that these phenotypic similarity measures were the most likely to capture functional relationships. The poor performance of character- or vector-based measures of phenotypic similarity lies at least in part in the fact that they can be misled by genes involved in the same function but having been assigned different phenotypic descriptions as for example positive and negative regulators having opposite effects on a particular cellular feature. These measures are also affected by differences in phenotypic annotations of any given genes across screens as, for example, they treat ‘metaphase delayed’ and ‘mitosis delayed’ as unrelated phenotypes of the same gene. Ontology-based semantic similarity measures on the other hand do not have this problem. Measures accounting for chance occurrence of a phenotype such as TF-IDF also perform better than the character-based methods and this could be attributed to the relationship between frequency of a phenotype and its specificity, i.e. more specific phenotypes tend to be less represented in the data. However, despite semantic similarity measures looking promising, only phenotypes with high semantic similarity in CMPO were associated with high functional similarity of GO cellular function annotations. To use this observation for automatically converting phenotypes into GO functional annotations, one would need to define a threshold of CMPO semantic similarity above which function assignment becomes reliable but how to select this threshold is unclear because it is liable to change when the ontology is expanded. Another downside is that only a small fraction of genes with phenotypes could be annotated with cellular functions in this way.

We therefore wondered if another approach could make better use of the information. Defining cellular functions as groups of co-occurring GO terms allowed us to recover a stronger link between phenotypic similarity and function. Conversely, defining phenotypes as sets of co-occurring CMPO terms allowed us to link these phenotypes to similar functions in GO. Therefore, with these definitions, similar cellular functions do lead to similar phenotypes and similar phenotypes are indicative of similar functions. Our results extend the observation by Glass and Girvan [28] that cancer signatures can associate with GO term communities but not branches of the Gene Ontology. We note that, by requiring as input a list of functionally-related genes, some network-based gene prioritization algorithms such as FUN-L [38] and GeneMANIA [39] implicitly rely on this definition of biological function and in light of our findings, this may contribute to their success in enriching candidate genes in the desired phenotypes.

Our observations have several practical implications. First, they suggest that clustering of phenotypic profiles using naive profile vector-based metrics (as commonly done in the field of RNAi screening) is sub-optimal for predicting the function of genes because these types of measures have low correlation with functional similarity but correlation can be improved by taking into account information content of the phenotypes. Instead of clustering the genes, we propose that a more meaningful approach would be to cluster the phenotypes based on the genes annotated with them and look for enrichment in functional terms in these clusters. Genes associated with a cluster of CMPO terms can then be annotated with the corresponding functional GO terms. This is relevant to any gene annotation task whether through curation of existing data or analysis of an RNAi screen with multiple phenotypes.

A second implication concerns the integration of phenotypic information with other biological data. Several candidate gene selection methods rely on the combination of multiple sources of information to increase accuracy and coverage of functional association between human genes. So far phenotypic data from RNAi screens have not been used in these data integration schemes. While supervised machine learning methods could learn to make functional annotations from phenotypic ones, the outcome critically depends on the quality of the training set which in turn depends on how one links functional annotations to phenotypes. This is important for example to design a relevant kernel for kernel-based methods such as support vector machines. In this context, the design of meaningful kernels for phenotypic similarity would be an advantage. In our experience, and consistent with results presented here, using standard metrics to compute similarity between phenotypic profiles leads to poor performance in retrieving functionally related genes. Our results suggests that better phenotype kernels could be derived by replacing individual phenotypes by clusters of CMPO terms derived from the annotation-based graph. In the same way, considering diseases as phenotypes, we suggest that functional similarity derived from the annotation-based clusters of GO terms could be more useful for predicting disease genes than semantic similarity-based functional similarity.

Finally, as not every single gene knock-down can reveal a phenotype, studies have turned to phenotyping genetic interactions using RNAi (e.g. [30, 40, 41]). Whether and how these can be integrated in the way we propose here is an area of future work.

## Conclusions

In this work we explored how gene phenotypic annotations from RNAi screens in human cells are related to functional annotations in GO. After selecting a relevant measure to compare phenotypic profiles, we compared gene pairs similarities using GO and CMPO and found that phenotypic similarity generally did not correlate with functional similarity in GO. However, redefining functions as groups of co-occurring GO terms allowed us to recover a stronger link between phenotypes and functions. Our observations are particularly relevant in situations where phenotypic similarities are used as a proxy for inferring gene functions such as in RNAi screen data analysis and curation, in integrating phenotypic data with other data and in the prediction of disease genes.

## Endnotes

^1^ http://www.geneontology.org/GO.evidence.tree.shtml.

^2^ http://www.ebi.ac.uk/cmpo.

^3^ http://geneontology.org/page/download-annotations.

^4^ http://www.ebi.ac.uk/fg/sym.

## Additional files

Additional file 1
**Table S1.** List of genes. (CSV 47 kb)

Additional file 2
**Figure S1.** Heatmap of the Gene x Phenotype matrix. (PNG 7680 kb)

Additional file 3
**Table S2.** Gene annotations to GO terms. (CSV 2048 kb)

Additional file 4
**Table S3.** Gene annotations to CMPO terms. (CSV 180 kb)

Additional file 5
**Figure S2.** Distribution of functional similarity in GO versus phenotypic similarity (and vice versa) for different levels of sparsity. (PDF 250 kb)

Additional file 6
**Figure S3.** Distribution of average semantic similarities between genes for those pairs with high phenotypic similarity (>6) after random assignment of GO similarity values. (PDF 45 kb)

## Abbreviations

AUC: Area under the ROC curve
CMPO: Cellular microscopy phenotype ontology
CPO: Cellular Phenotype Ontology
EMBL: European molecular biology laboratory
GO: Gene ontology
IC: Information content
IDF: Inverse document frequency
IEA: Inferred from electronic annotation
IMP: Inferred from mutant phenotype
OBO: Open biomedical ontologies
PCA: Principal component analysis
PCC: Pearson’s correlation coefficient
RNAi: RNA interference
ROC: Receiver operating characteristic
siRNA: small interfering RNA
TF-IDF: Term frequency - inverse document frequency
