# Erratum to: How can functional annotations be derived from profiles of phenotypic annotations?

**DOI:** 10.1186/s12859-017-1607-y

**Published:** 2017-03-27

**Authors:** Beatriz Serrano-Solano, Antonio Díaz Ramos, Jean-Karim Hériché, Juan A. G. Ranea

**Affiliations:** 1 Department of Molecular Biology and Biochemistry, University of Málaga, Boulevard Louis Pasteur, 29071 Málaga, Spain; 2 Department of Algebra, Geometry and Topology, University of Málaga, Boulevard Louis Pasteur, 29071 Málaga, Spain; 3 European Molecular Biology Laboratory, Meyerhofstrasse 1, 69117 Heidelberg, Germany; 4 CIBER de Enfermedades raras (CIBERER), Madrid, Spain

## Erratum

Upon publication of this article [1], it was brought to our attention that Table 1 was incorrectly presented. The correct Table 1 is shown below and has been updated in the original article.

## References

[1] Serrano-Solano B (2017). How can functional annotations be derived from profiles of phenotypic annotations?. BMC Bioinformatics.

