# Supplementary material for: How can functional annotations be derived from profiles of phenotypic annotations?
Source: BMC Bioinformatics. 2017 Feb 10;18:96. doi: 10.1186/s12859-017-1503-5 (PMC5304448; doi:10.1186/s12859-017-1503-5)

Figure S2. Distribution of functional similarity in GO versus phenotypic similarity (and vice versa) for different levels of sparsity.

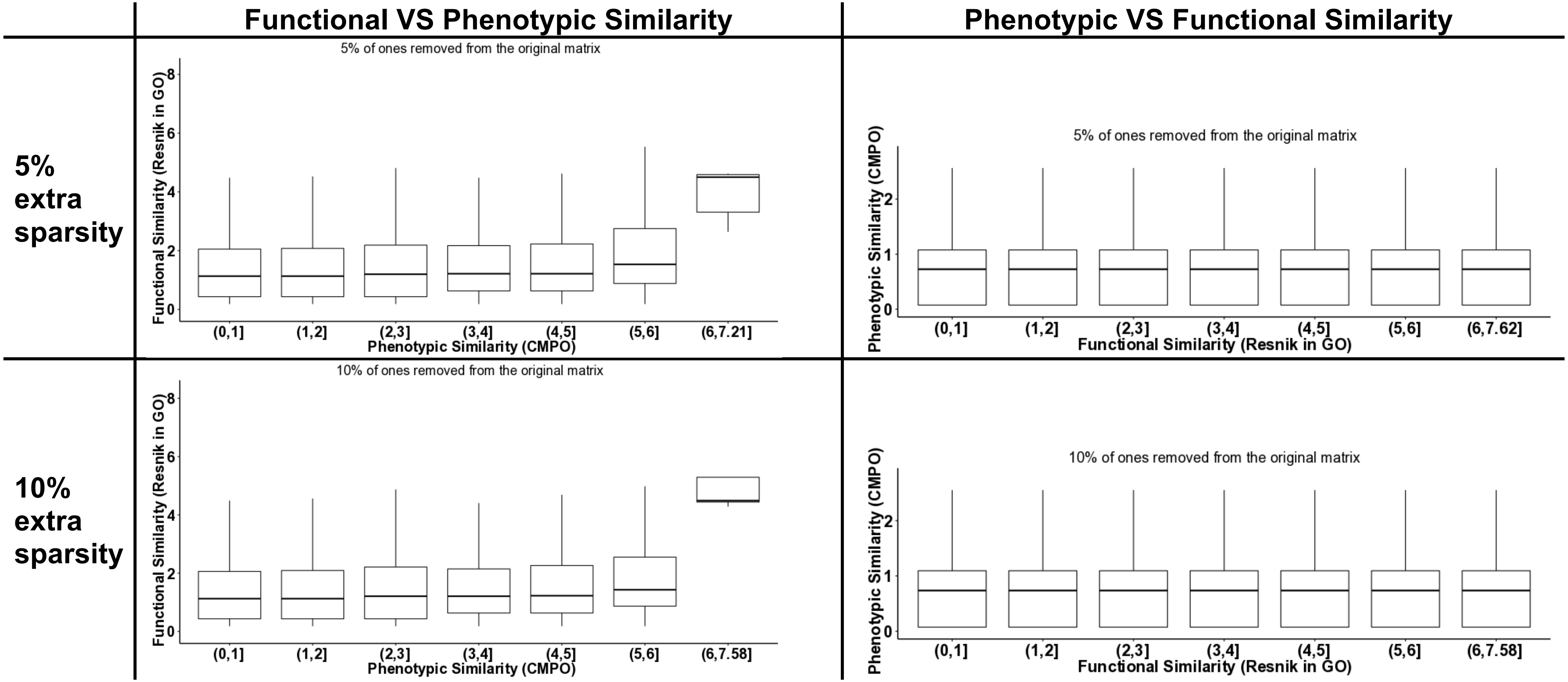

20% extra sparsity

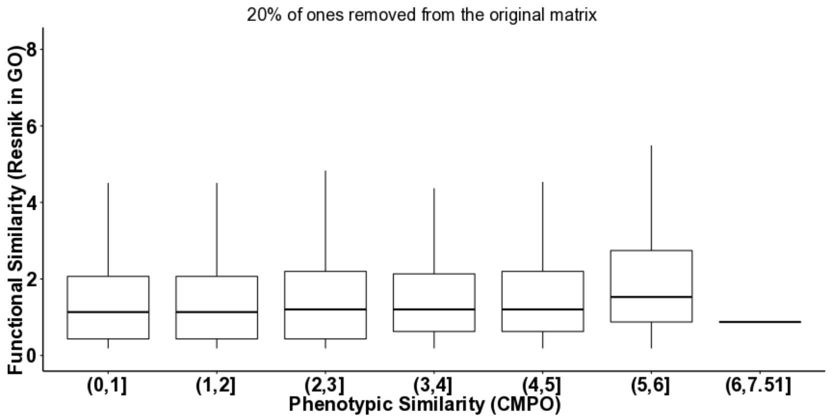

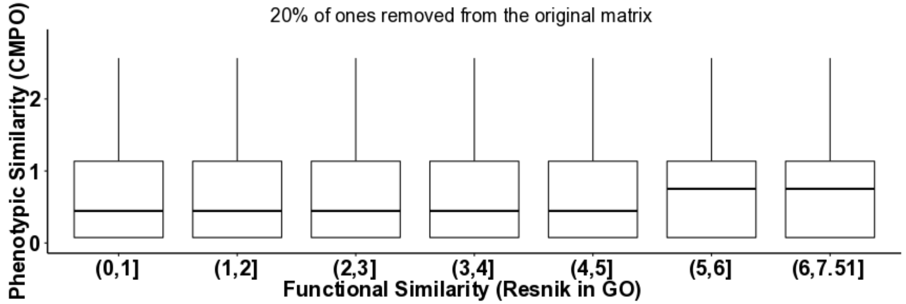

30% extra sparsity

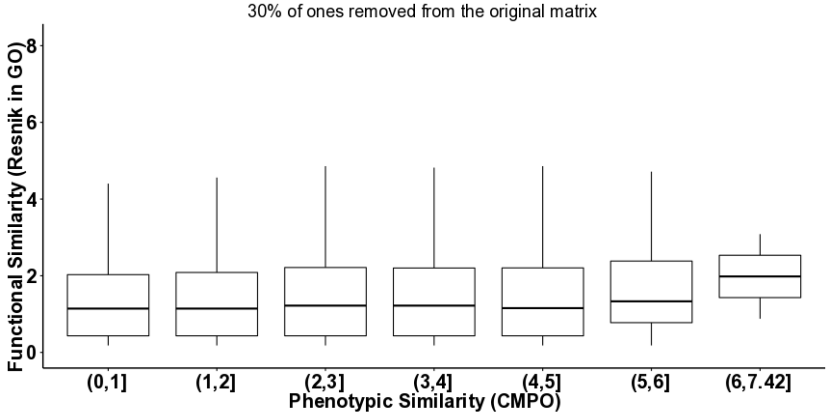

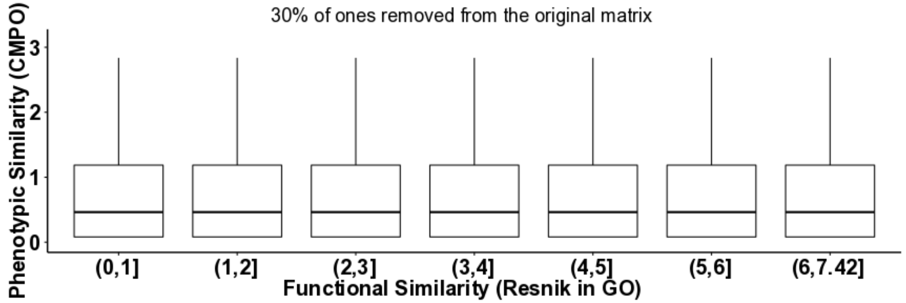

Supplement: Additional file 5 — Figure S2. Distribution of functional similarity in GO versus phenotypic similarity (and vice versa) for different levels of sparsity. (PDF 250 kb) [file 12859_2017_1503_MOESM5_ESM.pdf]
