# Supplementary material for: How can functional annotations be derived from profiles of phenotypic annotations?
Source: BMC Bioinformatics. 2017 Feb 10;18:96. doi: 10.1186/s12859-017-1503-5 (PMC5304448; doi:10.1186/s12859-017-1503-5)
Supplement: Additional file 6 — Figure S3. Distribution of average semantic similarities between genes for those pairs with high phenotypic similarity (>6) after random assignment of GO similarity values. (PDF 45 kb) [file 12859_2017_1503_MOESM6_ESM.pdf]

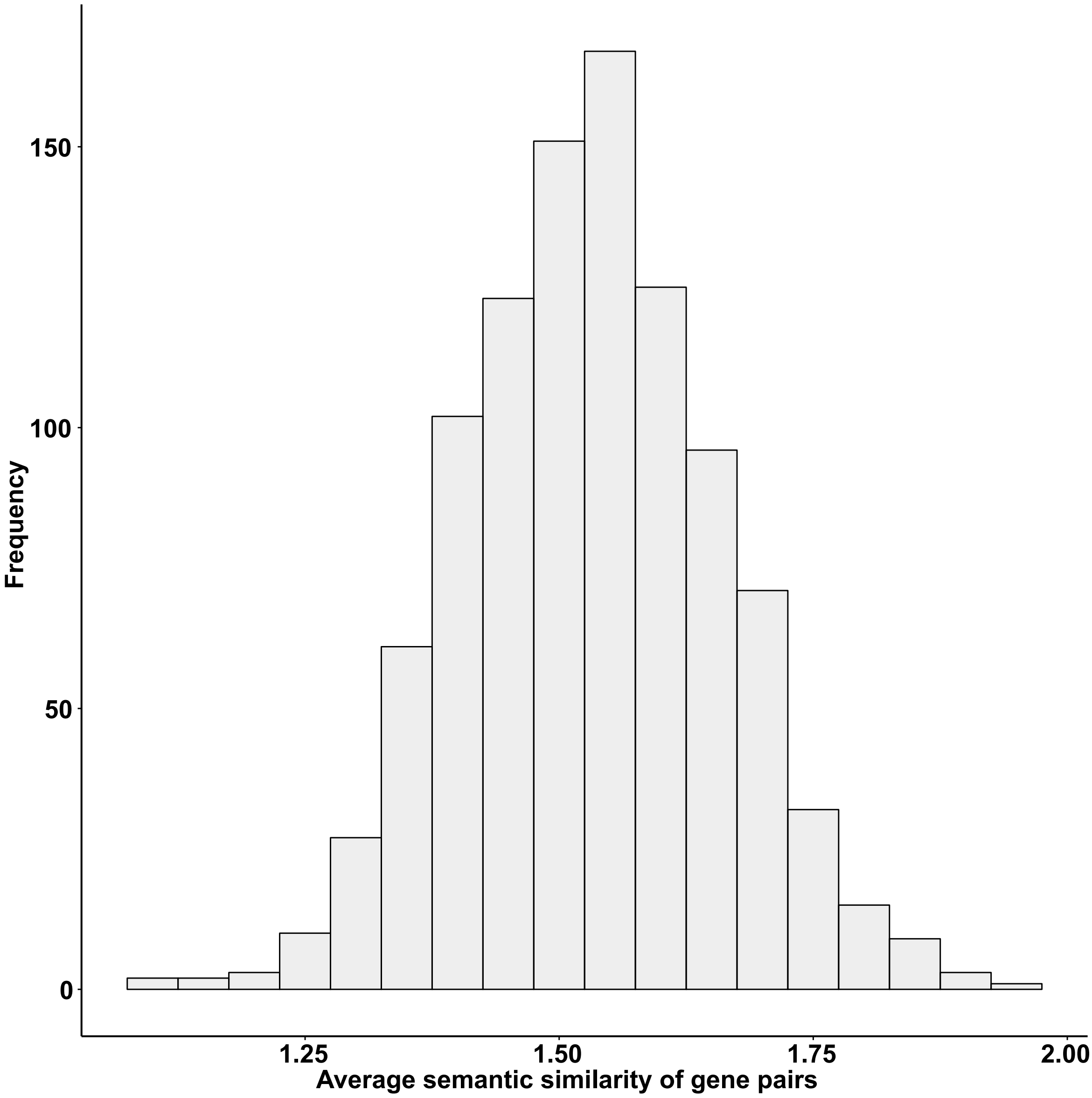

Figure S3. Distribution of average semantic similarities between genes for those pairs with high phenotypic similarity (>6) after random assignment of GO similarity values. The average of the random distribution is lower (1.53) than the one we observed (2.98).
